# Draft Genome Sequence of *Mycobacterium bohemicum* Strain DSM 44277^T^

**DOI:** 10.1128/genomeA.00878-15

**Published:** 2015-08-06

**Authors:** Shady Asmar, Michael Phelippeau, Catherine Robert, Olivier Croce, Michel Drancourt

**Affiliations:** Aix-Marseille Université, URMITE, CNRS, UMR 7278, IRD 198, Faculté de Médecine, Marseille, France

## Abstract

The *Mycobacterium bohemicum* strain is a nontuberculosis species mainly responsible for pediatric cervical lymphadenitis. The draft genome of *M. bohemicum* DSM 44277^T^ comprises 5,097,190 bp exhibiting a 68.64% G+C content, 4,840 protein-coding genes, and 75 predicted RNA genes.

## GENOME ANNOUNCEMENT

*Mycobacterium bohemicum* is a slow-growing, scotochromogenic mycobacterium characterized by biochemical inertia and a unique 16S RNA gene sequence (1). Although the first strain was isolated from the sputum of a 53-year-old patient with Down’s syndrome (1), most *M. bohemicum* isolates have been collected from pediatric cervical lymphadenitis (2–5). Furthermore, *M. bohemicum* has also been isolated from animal and inanimate environmental sources (6).

To facilitate the development of advanced molecular tools for the detection and identification of this species, whole-genome sequencing of *M. bohemicum* DSM 44277^T^ originally isolated from a sputum specimen was performed. Genomic DNA was isolated from *M. bohemicum* strain DSM 44277^T^ grown in MGIT Middlebrook liquid culture (Becton Dickinson, Le Pont-de-Claix, France) at 37°C in a 5% CO_2_ atmosphere. *M. bohemicum* genomic DNA was then sequenced in 2 Illumina MiSeq runs (Illumina, Inc., San Diego, CA, USA) using a 5-kb mate-paired library and one Roche 454 run using a 5-kb paired-end library (Roche, Basel, Switzerland). Reads from the 454 sequencer were assembled using Newbler software (Roche) and reads from Illumina were trimmed using Trimmomatic (7), and assembled using SPAdes v3.5 (8, 9). Contigs were combined together by SSPACE v2 (10), Opera v2 (11) helped by GapFiller v1.10 (12), and homemade tools in Python to refine the set. Finally, the draft genome of *M. bohemicum* DSM 44277^T^ consists of 18 scaffolds and 31 contigs containing 5,097,190-bp. The G+C content of this genome is 68.64%. Noncoding genes and miscellaneous features were predicted using RNAmmer (13), ARAGORN (14), Rfam (15), Pfam (16), and Infernal (17). Coding DNA sequences (CDSs) were predicted using Prodigal (18) and functional annotation was achieved using BLAST+ (19) and HMMER3 (20) against the UniProtKB database (21). The genome was shown to encode at least 75 predicted RNAs including three rRNAs, 57 tRNAs, one transfer-messenger RNA (tmRNA), and 14 miscellaneous RNAs. A total of 4,840 identified genes yielded a coding capacity of 4,578,093 bp (coding percentage, 89.82%). Among these genes, 255 (5.27%) were found to be putative proteins and 785 (16.22%) were assigned as hypothetical proteins. Moreover, 3,383 genes matched a least one sequence in the Clusters of Orthologous Groups database (22, 23) with BLASTp default parameters. *In silico* DNA-DNA hybridization (DDH) (24) was performed with nine reference genomes selected on the basis of their 16S rRNA gene proximity with *M. bohemicum*. The *M. bohemicum* genome was locally aligned 2-by-2 using the BLAT algorithm (25, 26) against each of the nine selected genomes, and DDH values were estimated from a generalized linear model (27). The DDH was of 27.7% (± 2.43) with *Mycobacterium avium* 104, 27.1% (± 2.42) with *Mycobacterium indicus* pranii MTCC 9506 and *Mycobacterium intracellulare* MOTT-02, 24% (± 2.39) with *Mycobacterium kansasii* ATCC 12478, 23.9% (± 2.39) with *Mycobacterium tuberculosis* H37Rv, 23.3% (± 2.38) with *Mycobacterium ulcerans* Agy99, 23.2% (± 2.38) with *Mycobacterium liflandii* 128FXT and *Mycobacterium asiaticum* DSM 44297, and 23.1% (± 2.37) with *Mycobacterium marinum* M. These data confirm that *M. bohemicum* is a unique species more closely related to the *M. avium* complex.

### Nucleotide sequence accession numbers.

The *M. bohemicum* strain DSM 44277^T^ genome sequence has been deposited at EMBL under the accession numbers CSTD01000001 to CSTD01000018.
